# The modulation of gut microbiota by herbal medicine to alleviate diabetic kidney disease – A review

**DOI:** 10.3389/fphar.2022.1032208

**Published:** 2022-11-14

**Authors:** Jinxin Du, Meina Yang, Zhongwen Zhang, Baorui Cao, Zhiying Wang, Jinxiang Han

**Affiliations:** ^1^ Shandong University of Traditional Chinese Medicine, Jinan, China; ^2^ NHC Key Laboratory of Biotechnology Drugs (Shandong Academy of Medical Sciences), Biomedical Sciences College, Shandong First Medical University, Jinan, China; ^3^ Shandong Key Laboratory of Rheumatic Disease and Translational Medicine, Department of Endocrinology and Metabology, The First Affiliated Hospital of Shandong First Medical University and Shandong Provincial Qianfoshan Hospital, Jinan, China

**Keywords:** gut-kidney axis, gut microbiota, diabetic kidney disease, herbal medicine, chronic kidney disease

## Abstract

The treatment of diabetic kidney disease (DKD) has been the key concern of the medical community. Herbal medicine has been reported to alleviate intestinal dysbiosis, promote the excretion of toxic metabolites, and reduce the secretion of uremic toxins. However, the current understanding of the modulation of the gut microbiota by herbal medicine to delay the progression of DKD is still insufficient. Consequently, we reviewed the knowledge based on peer-reviewed English-language journals regarding regulating gut microbiota by herbal medicines in DKD. It was found that herbal medicine or their natural extracts may have the following effects: modulating the composition of intestinal flora, particularly *Akkermansia*, *Lactobacillus*, and *Bacteroidetes*, as well as adjusting the F/B ratio; increasing the production of SCFAs and restoring the intestinal barrier; reducing the concentration of uremic toxins (p-cresol sulfate, indole sulfate, TMAO); inhibiting inflammation and oxidative stress.

## 1. Introduction

Diabetic kidney disease (DKD) is one of the most severe complications of diabetes mellitus (DM) and a significant cause of chronic kidney disease (CKD) and end-stage renal disease (ESRD). There is evidence that approximately 30–40% of diabetic patients will develop DKD (Gheith et al., 2016; Barutta et al., 2020). The prevalence of DKD is on the rise, along with DM, which results in enormous health care expenditures, making it a health problem and a social one (Williams et al., 2020). As a result, the treatment of DKD has been a significant concern in the medical community. The use of sodium-glucose cotransporter2 inhibitors and RAS inhibitors has been proven beneficial in patients with DKD (Saglimbene et al., 2018; van Baar et al., 2018). However, the risk of developing ESRD remains relatively high, which makes the need to explore new therapeutic approaches and targets particularly important.

Herbal medicines are commonly used to treat kidney disease in many countries, particularly China. It has been demonstrated in human clinical studies and animal experiments that herbal medicines protect kidney function. A systematic review and meta-analysis comprising 20 randomized controlled studies showed significant improvement in proteinuria and renal function due to herbal medicine treatment for DKD. The patients tolerated the treatment well, with a very low incidence of adverse events (Zhang et al., 2019). Nevertheless, the exact mechanism by which botanical drugs exert their therapeutic effects remains unclear. Among the possible mechanisms of action are the regulation of metabolic disorders, inflammatory response modulation, oxidative stress, antifibrosis, and regulation of microRNAs(Lu et al., 2019).

The gut microbiota plays an influential role in the dynamic homeostasis of health and disease by participating in immunity, metabolic regulation, and nutrient absorption (Kim et al., 2021). It is believed that the bidirectional relationship between the gut microbiota and the kidney (the gut-kidney axis) contributes to the pathogenesis of CKD (Monteiro and Berthelot, 2021). Several studies have confirmed intestinal microbiota and metabolite altered in CKD patients. Previous studies have shown that herbal medicines and their natural extracts may have specific protective effects on renal function. These effects can slow the progression of renal disease, but the exact mechanism is still not completely clear. It has been speculated that botanical drugs may exert their pharmacological effects by acting on the gut-kidney axis: modulating the gut microbiota, restoring the integrity of the intestinal barrier, and inhibiting inflammation. Based on a review of peer-reviewed English journals, we examined the role of intestinal microbiota in human physiopathology, the potential mechanisms of gut microbiota contributing to renal diseases, and the effects of botanical drugs and their active ingredients on retarding renal disease progression by affecting the gut-kidney axis.

## 2. Gut microbiota and metabolisms

### 2.1 Gut microbiota

It is well known that the human gastrointestinal tract has a surface area of approximately 250–400 m^2^, in which billions of trillions of microorganisms are colonized (Thursby and Juge, 2017). It has been reported that the amount of microorganisms in the human intestinal tract is approximately ten times greater than that of human cells (Zhu et al., 2010), which is 100 times wider than the genomes of the human body (Gill et al., 2006). A study identified 2172 prokaryotic species isolated from the human body and found that these species were derived from 12 different bacterial phyla. 93.5% of the bacteria isolated were from the phylum of *Firmicutes*, *Proteobacteria*, *Actinobacteria*, and *Bacteroidetes*, accounting for 31.1%, 29.5%, 25.9%, and 7.1%, respectively (Hugon et al., 2015).

The gut microbiota is closely associated with the host and can be considered a “vital organ" (Amon and Sanderson, 2017). In healthy individuals, the intestinal flora is in harmony with the host, a process known as symbiosis. Lifestyles and external factors (e.g., diet, disease, antibiotics), as well as geographical factors, are continuously influencing the composition and function of the gut microbiota. When this balance is disrupted, dysbiosis occurs. (Stavropoulou et al., 2020). It has been suggested that the gut microbiota is in constant communication with organs or systems such as the brain (Dinan and Cryan, 2017), kidney (Evenepoel et al., 2017), blood vessels (Karbach et al., 2016), and immune system (Takiishi et al., 2017). The gut microbiota plays a vital role in the dynamic homeostasis of health as well as the pathological state by participating in immunity, metabolic regulation, and nutrient absorption of the host (Kim et al., 2021).

### 2.2 Microbiota metabolism

The intestinal microbiota participates in various metabolic processes in the human body under physiological conditions. One of the most prominent functions is the fermentation of foods in the large intestine. In addition, the gut microbiota plays an essential role in the synthesis of vitamins (e.g., B and K) and essential amino acids (threonine, lysine, *etc.*), the production of short-chain fatty acids (SCFAs), as well as the stimulation of the immune system and the integrity of the intestinal barrier (Sonnenburg and Bäckhed, 2016; Yang et al., 2018).

#### 2.2.1 Short-chain fatty acids

Colonic SCFAs are composed primarily of acetate (60%), propionate (25%), and butyrate (15%), which have a variety of functions, such as regulating energy metabolism, maintaining the epithelial barrier, and mediating inflammation and immunity. (Cummings et al., 1987).

SCFAs constituted the primary energy source of the intestinal tract and provide 60–70% of the energy source for intestinal epithelial cells (Flint et al., 2012), which maintains the integrity of the epithelium. Studies have shown that SCFAs, particularly butyrate, are capable of maintaining the intestinal barrier by regulating the expression of tight junction proteins, which may be mediated by the activation of AMP-activated protein kinases or by the downregulation of claudin two expressions (Daly and Shirazi-Beechey, 2006; Peng et al., 2009). Moreover, acetate, propionate, and butyrate treatment ameliorated renal dysfunction upon renal ischemia-reperfusion in acute kidney injury models (Antza et al., 2018). SCFAs have also been found to be involved in the biosynthesis of mitochondria, which may have a positive effect on hypoxia in renal epithelial cells (Andrade-Oliveira et al., 2015). Additionally, SCFAs may exert their protective effects through their interaction with the G protein-coupled receptors GPR43 and GPR109a (Li et al., 2020).

#### 2.2.2 Uremic toxins

Proteins that reached the large intestine were degraded by intestinal flora into metabolites such as ammonium, amines, thiols, phenols, and indoles (Meijers et al., 2018). Some of the protein fermentation products have toxic effects and are called uremic toxins (Gryp et al., 2017), which played an essential role in the progression of DKD. P-cresol sulfate, indole sulfate, and trimethylamine N-oxide (TMAO) is the most representative uremic toxins of intestinal origin, with their precursors being p-cresol, indole and trimethylamine (TMA) respectively (Pahl and Vaziri, 2015; Fernandez-Prado et al., 2017; Gryp et al., 2020). Hepatic enzymes then transformed these metabolites into uremic toxins (Mosterd et al., 2021). Several studies have indicated that indole sulfate and p-cresol sulfate could compromise the kidney and cardiovascular system and thus contribute to the progression of CKD and cardiovascular complications (Liabeuf et al., 2010; Wu et al., 2011; Vanholder et al., 2014). Experimental studies have shown that indole sulfate and p-cresol sulfate, as protein-bound uremic toxins, can induce oxidative stress and promote tubulointerstitial fibrosis, resulting in persistent deterioration of renal function (Fukagawa and Watanabe, 2011). Indole sulfate could increase the oxygen consumption of the proximal tubule, thereby causing hypoxia in the kidney (Hasegawa et al., 2017). P-cresol sulfate has the capacity to alter insulin signaling through the activation of ERK1/2, which triggers insulin resistance in mice (Koppe et al., 2012).

As a flora-dependent product, TMAO is primarily eliminated by the kidney. With the progression of CKD, the kidney filtration function decreases with the accumulation of TMAO in the body. An increase in TMAO levels could lead to impaired glucose tolerance and elevated fasting blood glucose in mice (Gao et al., 2014; Fang et al., 2021), which was also observed in DM patients (Winther et al., 2019). The critical role of TMAO in the development of kidney disease has been demonstrated. A study of plasma metabolites in patients with CKD showed that there was a significant correlation between plasma TMAO with its precursor and glomerular filtration rates (Guo et al., 2021). Another study showed a positive correlation between plasma TMAO levels and serum creatinine, blood urea nitrogen, total 24-h UTP, and urine microprotein levels. HE staining showed that TMAO exacerbated renal tubular injury (Fang et al., 2021). Moreover, these manifestations, such as renal fibrosis and inflammation, can be inhibited by the TMA formation inhibitor 3, 3-Dimethyl-1-butanol (Sun et al., 2017).

## 3. Gut microbiota and metabolic characters in DKD

### 3.1 Gut microbiota changes in DKD

In the past decades, studies have demonstrated that alterations in the gut microbiota were correlated with metabolic disorders and closely involved in the progression of DM and DKD. In comparison with healthy subjects, DM patients exhibit a significant decrease in the relative abundance of gut microbiota (Larsen et al., 2010). A relatively high content of Gram-negative bacteria belonging to phylum *Bacteroidetes* and *Proteobacteria* in the intestine has been observed, whose main compounds of the bacterial outer membrane are lipopolysaccharides (LPS), which act as an inflammation stimulating factor and induce the aggregation of inflammation (Allcock et al., 2001). It has been observed that Changes in the *Firmicutes* to *Bacteroidetes* (F/B) ratio are correlated with blood glucose levels in DM (Li et al., 2020; Xia et al., 2021). In addition, studies have found a decreased level of *bifidobacteria* in patients with type 2 diabetes mellitus (T2DM), which can help with the maintenance of a well-connected epithelial barrier and has some anti-inflammatory effects (Sedighi et al., 2017). The altered microbiota of DKD patients is similar to that of DM patients; a study showed a much lower abundance and diversity of intestinal flora in DKD patients than that in healthy subjects. The amounts of *Actinobacteria*, *Bacilli*, *Coriobacteriia,* and *Negativicutes* in the intestine of DKD patients were higher than in the control group, whereas the abundance of *Alphaproteobacteria* and *Clostridia* was lower (Du et al., 2021).

Tao et al. (2019)found that the composition and abundance of gut microbiota in the DKD group were significantly different from both healthy and DM groups, with *Prevotella* able to distinguish DM patients from healthy subjects, while the distinction between DM and biopsy-proven DKD patients could be characterized by *Escherichia-Shigella* and *Prevotella*, which may represent a genus characteristic of the DKD microbiota. The gut microbiota changes during the progression of DKD as well. Chen et al. (2021)found a lower diversity of gut microbiota in the stage III DKD group than in the healthy group, DM group, as well as groups with other stages of DKD. They also found a positive correlation between *Alistipes* and 24-h urine protein quantity (24-h UTP), whereby a high level of 24-h UTP is an injurious factor. However, the specific role of these differential organisms in the pathogenesis of DKD still requires further exploration.

### 3.2 Microbiota metabolic characteristics in DKD

Patients with DKD may have a decreased production of SCFAs and retention of proteins along with their fermentation products due to controlled intake of potassium-containing diets, medications, dialysis, reduced gastrointestinal motility, and co-morbidities (Bammens et al., 2003; Friedman, 2009). As compared to the healthy group, patients in the DKD group had lower levels of serum acetic acid, propionic acid, butyric acid, and total SCFAs (Zhong et al., 2021; Cai et al., 2022). Patients with DM and DKD had similar metabolic changes. However, no relevant studies have been found regarding the metabolic dynamics of intestinal flora before, during, and after the onset of DKD in patients with DM.

DKD patients also generally exhibit altered protein metabolism in the colon. The shift in microbial metabolism from carbohydrate to protein metabolism in patients with CKD results in increased levels of protein fermentation end products (uremic toxins) in the plasma (Mosterd et al., 2021). It has been demonstrated that in ESRD patients, the species with butyrate-forming enzymes were significantly decreased, which resulted in higher levels of indole and p-cresol and lower levels of butyrate (Wong et al., 2014). Other studies have also confirmed the elevation of uremic toxins in the intestine of CKD patients due to dysbiosis (Meijers et al., 2009; Yang et al., 2021). The results of a systematic review confirmed the toxicity of indoles and p-cresol sulfates and their adverse effects on the progression of renal and vascular diseases (Vanholder et al., 2014). A meta-analysis that enrolled 11 studies showed that elevated indole sulfate and p-cresol sulfate levels in CKD patients were associated with an increased mortality rate (Lin et al., 2015). In addition, indole sulfate and p-cresol sulfate can induce epithelial-mesenchymal transition by activating the RAS system, which in turn promotes renal fibrosis (Sun et al., 2012; Lu et al., 2018).

It was found that TMAO levels were significantly higher in CKD patients than in healthy subjects (median plasma TMAO concentration, 30.33 μmol/L vs 2.08 μmol/L). Furthermore, a significant increase in TMAO levels was also observed in mice that received fecal transplants from CKD patients (Xu et al., 2017; Yang et al., 2021). Elevated TMAO levels in the circulating system could impair kidney function and lead to progressive renal fibrosis (Tang et al., 2015).

Due to the importance of the gut microbiota and its metabolites in the pathogenesis of DKD and CKD, several interventions for treating DKD have been developed by modifying the gut microbiota and microbiota metabolites. These interventions include dietary modification (Li et al., 2020), resistant starch (Snelson et al., 2019), probiotics (Hsiao et al., 2021), prebiotics (Koppe and Fouque, 2017), synbiotics (Rossi et al., 2016), and some adsorbents (Schulman et al., 2015), which have also been shown to be useful to protect renal function.

## 4. Herbal medicine, gut-kidney axis, and dkd/ckd

Herbal medicine has been shown to have positive effects on the treatment of DKD in clinical studies. With the increasing understanding of the importance of gut microbiota and their metabolites in the pathogenesis of CKD and DKD, several studies have begun to explore whether herbal medicines (Table1) and their natural extracts (Table2) could exert renoprotective effects by affecting the gut microbiota and their metabolites through the gut-kidney axis.

**TABLE 1:** Summary of the effects of herbal medicine on gut microbiota and laboratory indexes.

Botanical drugss/Herbal pairs/Formulas	Subjects	Effects on gut microbiota	Primary outcomes	References
Rheum palmatum L	5/6 nephrectomy rats	Decreased *Akkermansia*, *Methanosphaera*, and Clostridiaceae	Decreased Scr, IL-1b and IL-6 levels	Ji et al. (2020)
		Increased *bacteroidetes*, *bacteroidales*, *bacteroidia*, *prevotella* et al	Decreased serum TMAO, TMA, IL-6, TNF-ɑ, IFN-y levels; reduced urine output	Ji et al. (2021)
		Increased *akkermansia-muciniphila*, *lactobacillus-aci-dophilus*, *bacteroides-caccae*, and *faecalibaculum-rodentium*	Reduced Scr, inflammation levels, improved kidney pathology	Ji et al. (2022)
Poria cocos	5/6 nephrectomy rats	Ameliorated microbial dysbiosis	Improved Ccr, lowered blood pressure, Scr, urea concentrations, and proteinuria	Feng et al. (2019)
Morus alba L	Streptozotocin and high-fat diet-induced DN rats	Increased *Bacteroidetes*, *Proteobacteria,* and *Clostridia*	Reduced FBG and urine glucose levels; enhanced insulin sensitivity; alleviated proteinuria and chronic renal damages	Yao et al. (2018)
Rehmannia glutinosa (Gaertn.) DC. and Cornus officinalis Siebold and Zucc	Adenine-induced CKD rats	Increased Ruminococcaceae *UCG-014*, *Ruminococcus 1*, *Pre-votellaceae_NK3B31_group*, Lachnospiraceae *NK4A136 group* and Lachnospiraceae *UCG-001*; decreased *Desulfovibrio*	Decreased 24 h urine protein; improved inflammation and hyperplasia of fibrous	Zhang et al. (2021)
*Astragalus* mongholicus Bunge and Salvia miltiorrhiza Bunge	Cyclosporin A-induced chronic nephrotoxicity mice	Modified the ratio of *Firmicutes* to *Bacteroidetes*, increased abundance of *Lactobacillus* and *Akkermansia*	Decreased IL-6, Scr, BUN, and UA; improved the pathology of kidney and colon	Han et al. (2021)
Scutellaria baicalensis Georgi and Styphnolobium japonicum (L.) Schott	Hypertensive Nephropathy rats	Decreased *Firmicutes/Bacteroidetes* ratio and Clostridiaceae, increased *Lactobacillus*	Decreased the blood pressure; ameliorated renal structure damage; decreased the levels of Cr, BUN, and mALB	Guan et al. (2021)
Shenyan Kangfu tablet	db/db mice	Decreased *Bacteroidetes*, increased *Firmicutes*	Reduced stimulated blood glucose and HbA1c levels, alleviated renal dysfunction, glomerular and tubular damage, and renal inflammation (TNF-α and IL-1β)	Chen et al. (2021)
Tangshen formula	Streptozotocin injection and uninephrectomy-induced DN rats	Increased the abundance of *bifidobacteria*, reversed the increased *Bacteroidetes-*to*-Actinobacteria* ratio	Reduced levels of indoxyl sulfate and metabolic endotoxemia/Lipopolysaccharide; decreased MCP-1 and TNF-α	Zhao et al. (2020)
QiDiTangShen granules	db/db mice	Decreased *Lactobacillus*, *Bacteroides,* and *Lachnospir-aceae_NK4A136_group*, increased *Alloprevotella*	Reduced urinary albumin excretion and attenuated the pathological injuries of the kidney; decreased serum levels of total bile acid and bile acid profiles	Wei et al. (2021)
Shenqi Yanshen Formula	Adenine-induced mice	Increased *f_*Succinivibrionaceae and *o_Aeromonadales*	Decreased Scr and BUN levels; reduced the degree of renal fibrosis; reduced TNF-α, IL-1β, and IL-6 expression	Zhang et al. (2022)
Qing-Re-Xiao-Zheng Formula	Streptozotocin and high-fat diet-induced DN mice	Increased Rikenellaceae and *Akkermansia*	Decreased urinary albumin, serum cholesterol, and triglycerides levels; attenuated renal injuries; suppressed TLR4 and NF-κB expression	Gao et al. (2021)
Jianpi Yishen decoction	5/6 nephrectomy rats	Increased butyrate-producing bacteria such as *Phascolarctobacterium*, *Coprococcus*, decreased *Clostridium_XIVb*	Decreased the levels of BUN, U-ALB, and TNF-a, improved kidney function	Zheng et al. (2020)
Sanhuang Yishen capsule	Streptozotocin and high-fat diet-induced DN rats	Increased *Lactobacillus*, Ruminococcaceae *UCG-005*, *Allobaculum*, *Anaerovibrio*, *Bacteroides* and *Candidatus_Saccharimonas*	High-dose SHYS reduced the serum levels of Cr, BUN, and 24-h urine protein; The middle-dose SHYS reduced 24-h urine protein and serum BUN levels; reduced oxidative stress, and inflammatory response	Su et al. (2022)

Abbreviations: Cr, creatinine; Scr, Serum creatinine; Ccr, creatinine clearance; BUN, blood Urea Nitrogen; UA, uric acid; mALB, microalbumin; U-ALB, U-mAlb, urine microalbumin; ACR, Urine albumin/creatinine.

**TABLE 2:** Summary of the effects of natural extracts on gut microbiota and laboratory indexes.

Ingredients	Subjects	Effects on gut microbiota	Primary outcomes	References
Emodin	5/6 nephrectomy rats	Increased *Lactobacillus*; reduced *Enteroroccus*, *Escherichia coli,* and *C. perfringens*	Reduced urea concentrations and urinary protein excretion; decreased urea and indoxyl Sulfate levels; improved renal function	Zeng et al. (2016)
Emodin-NP	5/6 nephrectomy rats	Returned the microbial balance, increased butyrate-producing bacteria	Reduced IL-1β, IL-6, and LPS levels in serum; improved intestinal barrier functions, downregulated TLR4, MyD88, and NF-κB expression in intestinal TLR4 signaling pathway; Improved renal function and inhibited tubulointerstitial injury	Lu et al. (2021)
Resveratrol	db/db mice	Increased *Bacteroides*, *Alistipes*, *Rikenella*, *Odoribacter*, *Parabacteroides*, and *Alloprevotella* abundance	Decreased Scr, blood urea nitrogen, and urine 24-h microalbuminuria levels; improved intestinal barrier function; ameliorated intestinal permeability and inflammation	Cai et al. (2020)
Resveratrol butyrate ester (RBE)	Adenine-fed rats	High-dose RBE increased the abundance of *Akkermansia*, *Blautia*, and *Enterococcus*	Reduced renal expression of GPR41 and Olfr78 protected adenine-treated rats against hypertension and renal dysfunction	Hsu et al. (2022)
Punicalagin	High-fat diet-induced mice	Increased SCFAs producing bacteria *Akkermansia*, *Eubacterium_coprostanoligenes_group* and Lachnospiraceae	Decreased CREA, UA, and BUN levels; ameliorated kidney architecture and function	Hua et al. (2022)
Rehmannia glutinosa leaves total glycoside	db/db mice	Increased Erysipelotrichaceae and *Acetatifacto*r	Decreased blood sugar and lipid levels as well as BUN, mALB, and Scr levels; alleviated pathological changes	Xu et al. (2020)
Total flavones of Abelmoschus manihot	Uninephrectomy, potassium oxonate, and proinflammatory diet-induced rats	Decreased *Bacteroidales* and *Lactobacillales* and increased *Erysipelotrichales*	Inhibited IL1b, TNF-α, NF-κB; decreased BUN, Scr, and SUA	Tu et al. (2020)
Curcumin	T2DM and DN patients	Increased *Bacteroides Bifidobacterium* and *Lactobacillus*	Attenuated U-mAlb excretion, reduced plasma MDA as well as LPS content; increased IκB	Yang et al. (2015)
Fisetin	Potassium oxonate and adenine-induced mice	Decreased *Firmicutes*, increased *Bacteroidetes* and *Epsilonbacteraeota*	Reduced serum uric acid, Scr, and BUN levels	Ren et al. (2021)
Bupleurum polysaccharides	Streptozotocin induced mice	Increased *Candidatus_Arthromitus*; BCP increased Rikenellaceae, *Ruminococcus*, *Oscillospira,* and *Roseburia*; BPs increased *Helicobacte*r and *Eubacterium*	Decreased blood glucose, cr and U-ALB; reduced TNF-α, IL-6; improved gut barrier	Feng et al. (2019)
Cordyceps cicadae polysaccharides	Streptozotocin and high-fat diet-induced DN rats	Increased *Verrucomicrobia*, *Actinobacteria, et al.*, decreased *Proteobacteria,* and *Deferribacteres* et al	Reduced collagen I, fibronectin, andα-SMA; decreased TNF-α, IL-1β, and IL-6; alleviated insulin resistance; decreased 24 h urine volume, urine protein, ACR, BUN and Scr, increased urine creatinine and Ccr	Yang et al. (2020)

Abbreviations: Cr, creatinine; Scr, Serum creatinine; Ccr, creatinine clearance; BUN, blood Urea Nitrogen; UA, uric acid; mALB, microalbumin; U-ALB, U-mAlb, urine microalbumin; ACR, Urine albumin/creatinine.

### 4.1 Botanical drugs

#### 4.1.1 Rheum palmatum L

The main official parts of Rheum palmatum L. are the root and rhizome. Research has shown that Rheum palmatum L. has several pharmacological properties such as anti-inflammatory, antioxidant, and most importantly, it protects against renal fibrosis (Wang et al., 2022). Rheum palmatum L. can also decrease the abundance of conditionally pathogenic microbes such as Alcaligenaceae, *Methanosphaera*, and Clostridiaceae in the intestine of 5/6 Nephrectomy rats compared to the CKD model, restoring the functions of the intestinal barrier and alleviating renal fibrosis (Ji et al., 2020). Ji et al. (Ji et al., 2021)further investigated the relationship between Rheum palmatum L. enema and TMAO. 5/6 Nephrectomy rats were divided into the model group, Rheum palmatum L. high-dose group (2.10 g/kg/day), and Rheum palmatum L. low-dose group (1.05 g/kg/day). After 8 weeks of enema treatment, renal interstitial fibrosis and tubular atrophy were significantly reduced in both high-dose, and low-dose group, serum levels of TMAO and TMA as well as inflammatory factors such as IL-6, TNF-ɑ, IFN-y was found to be decreased. It may be related to the increase in the abundance of commensal organisms and the decrease in the abundance of conditionally pathogenic species. Another study by the same research team found that Rheum palmatum L. enema (0.2 g/ml×5 ml) increased the content of SCFAs in the intestine of 5/6 Nephrectomy rats by increasing the abundance of SCFA-producing flora such as *Akkermansia-muciniphila*, *lactobacillus-acidophilus*, *Bacteroides-caccae*, and *Faecalibaculum-rodentium*, thereby regulating tight junction proteins and repairing the damaged intestinal barrier, which reduced inflammation and improved kidney functions (Ji et al., 2022). In the three studies described above, Rheum palmatum L. granules were used. Due to the fact that its extraction process is unknown, it has yet to be determined whether granules can replace the efficacy of expressed Rheum palmatum L. The use of aqueous decoctions in conjunction with UPLC analysis may be considered in future studies.

#### 4.1.2 Poria cocos

Poria cocos is a fungus; its sclerotium, called fu-ling, has a long history of pharmaceutical use. Modern studies have shown that Poria cocos have anti-inflammatory, antioxidant, anti-fibrotic, and nephroprotective effects (Ríos, 2011). As reported by Feng et al. (2019), Poria cocos (250 mg/kg/day) and periodic acid A (a component of Poria cocos, 10 mg/kg/day) were able to ameliorate flora dysbiosis, protect the epithelial barrier, and retard renal fibrosis in 5/6 nephrectomy rats. Additionally, both of them improved endogenous creatinine clearance, decreased blood creatinine and urea nitrogen concentrations, and reduced proteinuria in rats. However, the study did not demonstrate the specific changes in the flora after PC and PCA treatments. Further study is necessary to confirm the changes in the flora.

#### 4.1.3 Morus alba L

Morus alba L. is a botanical drug with a long history of application and contains abundant active ingredients, including polyphenols and polysaccharides. The role of Morus alba L. in DKD treatment has also been investigated. Treatment with Morus alba L. reduced blood glucose and urinary protein levels, improved insulin resistance, and attenuated renal tubular interstitial fibrosis in high-fat diet (HFD) and streptozotocin (STZ) induced rats (Yao et al., 2018). These effects may be attributed to the fact that Morus alba L. increased the *Bacteroidetes* and *Proteobacteria* phylum in the intestine of DKD rats, with a decrease in the former phylum associated primarily with hyperglycemia, whereas the proportion of *β-Proteobacteria* in the latter phylum was found to be positively associated with blood glucose levels (Larsen et al., 2010). Since Morus alba L. was administered as a powder mixed with conventional feed, this study’s specific dose of administration was not described. It may be advantageous to utilize extracts or aqueous decoctions in future studies.

### 4.2 Herbal pairs

#### 4.2.1 Rehmannia glutinosa (gaertn.) DC. And cornus officinalis Siebold and Zucc

The main official part of Rehmannia glutinosa (Gaertn.) DC. is the dried rhizome, while the central official part of Cornus officinalis Siebold and Zucc. Is the mature dried fruit pulp. Both of them have been used for thousands of years as a standard pair in Chinese herbal formulas. Modern pharmacological studies have shown that they have some anti-inflammatory and anti-diabetic effects (Liu et al., 2017; Ye et al., 2020) and that the Rehmannia glutinosa (Gaertn.) DC. and Cornus officinalis Siebold and Zucc. Pair can additionally be used in the treatment of CKD. A study conducted by Zhang et al. (2021) found that treatment with Rehmannia glutinosa (Gaertn.) DC. and Cornus officinalis Siebold and Zucc. Mixture (1:2, 3.75 g/kg/d) for 14 days improved the abundance of gut microbiota in adenine-induced CKD rats compared to the model group. Which is characterized by an increase in potentially nephroprotective beneficial species such as Ruminococcaceae *UCG-014*, *Pre-votellaceae_NK3B31_group*, and Lachnospiraceae *UCG-001*, as well as a decrease in pathogenic bacteria such as *Desulfovibrio* that may damage the intestinal epithelial barrier. This also indicates that Rehmannia glutinosa (Gaertn.) DC. and Cornus officinalis Siebold and Zucc. Pair may be capable of protecting against CKD by modulating the gut microbiota and affecting the production of uremic toxins. The efficacy of Rehmannia glutinosa (Gaertn.) DC. and Cornus officinalis Siebold and Zucc. The paired application was also found to be superior to that of Rehmannia glutinosa (Gaertn.) DC (3.75 g/kg/d) or Cornus officinalis Siebold and Zucc (3.75 g/kg/d) alone in terms of the restoration of the flora.

#### 4.2.2 *Astragalus* mongholicus Bunge and Salvia miltiorrhiza bunge

The main official part of *Astragalus* mongholicus Bunge is the root, and the medicinal parts of Salvia miltiorrhiza Bunge are the root and rhizome. As a combination, they can alleviate kidney damage and protect kidney functions in the treatment of CKD (Huang et al., 2018). Studies have found that the combination of *Astragalus* mongholicus Bunge and Salvia miltiorrhiza Bunge (2:1, 8.4 g/kg/d) restored the abundance and diversity of intestinal flora in cyclosporin A-induced mice and modified the ratio of *Firmicutes* to *Bacteroidetes* (F/B), thereby attenuating the disorder of intestinal flora to some extent (Han et al., 2021). In addition, the increased abundance of *Lactobacillus* and *Akkermansia*, which serve as lactic and butyric acid-producing probiotics, respectively, can alleviate renal fibrosis by modulating bacterial metabolites to ameliorate renal inflammation, reduce uremic toxins production, and restore the intestinal barrier (Belzer et al., 2017; Lopes et al., 2018). Furthermore, similar results were obtained in cyclosporin A-induced mice that received fecal transplantation from *Astragalus* mongholicus Bunge and Salvia miltiorrhiza Bunge-treated mice (Han et al., 2021).

#### 4.2.3 cutellaria baicalensis Georgi and Styphnolobium japonicum (L.) schott

The main official part of Scutellaria baicalensis Georgi is the root, while the main official part of Styphnolobium japonicum (L.) Schott is the flower. The Scutellaria baicalensis Georgi and Styphnolobium japonicum (L.) Schott are both traditional Chinese medicines with various pharmacological properties that can also be used to alleviate kidney injuries. Guan et al. (2021)found that Scutellaria baicalensis Georgi (0.9 g/kg/d) treatment increased intestinal *Prevotella-9* and *Akkermansia* abundance in spontaneously hypertensive rats, while Styphnolobium japonicum (L.) Schott (0.9 g/kg/d) treatment increased the abundance of *Corynebacterium* and *Prevotella-9*. The Scutellaria baicalensis Georgi and Styphnolobium japonicum (L.) Schott combination (0.9 g/kg/d each) increased *Lactobacillus* and decreased Clostridiaceae, while the former was associated with tight junction expression as well as intestinal permeability and the latter with indole production (Niwa, 2013; Robles-Vera et al., 2018). A decreased F/B ratio was also observed after Scutellaria baicalensis Georgi and Styphnolobium japonicum (L.) Schott combined treatment. It was hypothesized that the combination of Scutellaria baicalensis Georgi and Styphnolobium japonicum (L.) Schott could improve dysbiosis, promote the production of SCFAs, decrease the production of indoles, inhibit inflammation and oxidative stress, and thus ameliorate hypertension-induced renal injuries.

### 4.3 Herbal formulas

#### 4.3.1 Shenyan Kangfu tablet

Shenyan Kangfu tablet (SYKFT) is a Chinese medicinal formula consisting of 13 botanical drugs that can be used to treat DKD (Chen et al., 2021), which includes Panax quinquefolius L (17.4 g), Panax ginseng C.A.Mey (5.8 g), Rehmannia glutinosa (Gaertn.) DC (58.1 g), Eucommia ulmoides Oliv (34.9 g), *Dioscorea* oppositifolia L (58.1 g), Salvia miltiorrhiza Bunge (29.1 g), Leonurus artemisia (Lour.) S.Y. Hu (58.1 g), Smilax glabra Roxb (58.1 g), Old-enlandia diffusa (Willd.) Roxb (29.1 g), *Glycine* max (L.). Merr (58.1 g), Imperata cylindrica (L.). Raeusch (87.2 g), Alisma plantago-aquatica L (29.1 g), and Platycodon grandiflorus (Jacq.) A. DC (58.1 g). Clinical studies have confirmed the safety and efficacy of SYKFT in the treatment of DKD (Kou et al., 2014). Further study by Chen et al. (2021) found that SYKFT (both 2 g/kg/d and 1 g/kg/d) could downregulate the abundance of phylum *Bacteroidetes*, elevate the abundance of phylum *Firmicutes*, reduce glycated hemoglobin and fasting glucose levels as well as urinary microprotein, improve renal thylakoid expansion and inflammatory response in db/db mice, resulting in a series of protective effects against DKD. They proposed that the renal inflammation in DKD is associated with changes in the gut microbiota and that the anti-DKD effect of SYKFT may be related to the regulation of the flora. The mechanisms by which SYKFT exerts its renal protective effect may be associated with the modulation of the gut microbiota and related proteins. It was not reported whether the effects of different doses of STKFT on gut microbiota were different, despite the study being designed with different doses of STKFT.

#### 4.3.2 Tangshen Formula

Tangshen Formula (TSF) is a Chinese medical formula used to treat DKD, which is found to improve glomerular filtration rate and reduce urinary protein in DKD patients (Li et al., 2015). There are seven ingredients in TSF, including *Astragalus* mongholicus Bunge, Euonymus alatus (Thunb.) Siebold, Rehmannia glutinosa (Gaertn.) DC., Citrus × aurantium L., Cornus officinalis Siebold and Zucc., Rheum palmatum L., and Panax notoginseng (Burkill) F.H.Chen (in the ratio of 10:5:4:3.4:3:2:1). Zhao et al. (2020), on the other hand, demonstrated the therapeutic effects of TSF *via* the regulation of gut microbiota and their metabolites. TSF (1.36 g/kg/d) significantly increased the abundance of *bifidobacterial* and reversed the increased *Bacteroidetes*-to-*Actinobacteria* ratio in STZ-induced DKD rats. It is believed that the levels of indole sulfate and LPS in the intestine were reduced by altering gut microbiota composition, resulting in reduced renal inflammation in DKD rats. The study findings would be enhanced if a positive control group could be established.

#### 4.3.3 Qidi Tangshen granules

Qidi Tangshen granules (QDTS) are composed of seven herbal medicines, including Rehmannia glutinosa (Gaertn.) DC., *Astragalus* mongholicus Bunge, Euryale ferox Salisb., Cornus officinalis Siebold and Zucc., Hirude nipponica Whitman, Rheum palmatum L., and Scleromitrion diffusum (Willd.) R.J.Wang (Gao et al., 2018). QDTS has been shown to be able to counteract kidney injuries and reduce proteinuria in DKD mice (Wang et al., 2019). A study further investigated the mechanisms of action of QDTS and concluded that it could modulate the gut microbiota composition of db/db mice (Wei et al., 2021). It was manifested by a reduced abundance of *Lactobacillus*, *Bacteroides,* and *Lachnospir-aceae_NK4A136_group* after QDTS treatment (3.37 g/kg/day), which according to their analysis, were positively correlated with indicators of kidney damage. An increment in the abundance of *Alloprevotella* has also been observed, which can produce SCFAs. Additionally, there was an improvement in the profile of bile acids. But there is no indication of the ratios of the seven components of the QDTS aqueous extract in the paper. It has been suggested that QDTS may exert its anti-DKD effects *via* the gut microbial-bile acid axis.

#### 4.3.4 Shenqi Yanshen formula

In the Shenqi Yanshen formula (SQYSF), there are seven natural Chinese medicinal drugs, including Panax ginseng C.A.Mey., *Astragalus* mongholicus Bunge, Rheum palmatum L., Epimedium sagittatum (Siebold and Zucc.) Maxim., Ligusticum striatum DC., Rehmannia glutinosa (Gaertn.) DC. and vinegar-processed carapax trionycis (Zhang et al., 2022). It was shown that SQYSF (3.6 g/kg/d) significantly reduced the blood creatinine and urea nitrogen levels in adenine-induced CKD mice, and HE staining showed improvement in renal fibrosis. The expression of inflammatory factors such as TNF-α, IL-1β, and IL-6 was also significantly reduced, which may be related to the increased abundance of *f_*Succinivibrionaceae and *o_Aeromonadales* in the intestine (Zhang et al., 2022). The article does not describe the specific ratios of the components in the SQYSF used, and an HPLC analysis may be required to clarify the major components.

#### 4.3.5 Qingre Xiaozheng formula

Qingre Xiaozheng formula (QRXZF) is formulated based on TCM theory for the treatment of DKD. According to Gao et al. (2021), QRXZF is composed of *Astragalus* mongholicus Bunge, Angelica Sinensis (Oliv.) Diels, Concha Ostreae, Rheum palmatum L., and four other botanical drugs (not mentioned in the paper). They found that QFXZF could reduce urinary protein and cholesterol levels in DKD mice. Moreover, they also noticed that QFXZF (15.6 g/kg/d) could increase the abundance of Rikenellaceae and *Akkermansia* in the intestine of HFD and STZ-induced DKD mice. The former could promote the production of SCFAs, while the latter could reduce intestinal mucosal damage and decrease serum LPS levels. Therefore, QRXZF may exert its pharmacological effects by regulating the disturbed intestinal microbiota, protecting the integrity of the intestinal barrier, and thereby decreasing serum LPS and relieving kidney inflammation. There are, however, few relevant studies on this formula. HPLC or UPLC analysis, as well as positive control group settings, may be required in order to enrich this study.

#### 4.3.6 Jianpi Yishen decoction

Jianpi Yishen decoction (JPYS) is a formula mainly used to treat CKD composed of *Astragalus* mongholicus Bunge, Atractylodes macrocephala Koidz., *Dioscorea* oppositifolia L., Cistanche deserticola Ma, Wurfbainia compacta (Sol. Ex Maton) Skornick. and A.D.Poulsen, Salvia miltiorrhiza Bunge, Rheum palmatum L. and *Glycyrrhiza* glabra L. in a 30, 10, 30, 10, 10, 15, 10, 6 g respectively. Zheng et al. (2020) found that JPYS (10.89 mg/kg/d) could restore the intestinal flora of 5/6 nephrectomy CKD rats, characterized by increased butyrate-producing bacteria such as *Phascolarctobacterium* and *Coprococcus* and decreased conditionally pathogenic bacteria represented by *Clostridium_XIVb*. Additionally, it resulted in an improvement in renal function and restoration of the blood reticulocyte count and blood calcium level in CKD rats.

#### 4.3.7 Sanhuang Yishen capsule

Sanhuang Yishen capsule (SHYS) is an herbal formula for the treatment of chronic kidney diseases, including DKD and Ig A nephropathy, which is composed of *Astragalus* mongholicus Bunge (15 g), Panax quinquefolius L (12 g), *Dioscorea* oppositifolia L (12 g), Cornus officinalis Siebold and Zucc (12 g), Cuscuta Chinensis Lam (12 g), Polygonatum sibiricum Redouté (12 g), Rehmannia glutinosa (Gaertn.) DC (15 g), Euryale ferox Salisb (12 g), Rosa laevigata Michx (12 g), Leonurus japonicus Houtt (10 g), Salvia miltiorrhiza Bunge (12 g), Conioselinum anthriscoides (H.Boissieu) Pimenov and Kljuykov (12 g), Atractylodes lancea (Thunb.) DC (10 g), Paeonia lactiflora Pall (10 g). And Gypsophila Vaccaria (L.) Sm (6 g). A previous study suggested that SHYS has some protective effects on DKD (Xiu-Hai et al., 2011). A study further explored the relationship between SHYS (1.62 g/kg/d) in regulating gut microbiota and renoprotective effects. They found that SHYS was able to reduce the F/B ratio and increase the abundance of beneficial bacteria such as *Allobaculum*, Ruminococcaceae *UCG-005*, and *Lactobacillus* in the intestine of HFD and STZ-induced DKD rats, which may be a possible mechanism for its nephroprotective effect. In addition, the combined analysis of untargeted metabolomics and 16 S rRNA sequencing results revealed that the altered gut microbiota might be related to metabolic processes such as the tricarboxylic acid cycle, arginine biosynthesis, and tyrosine metabolism (Su et al., 2022).

#### 4.3.8 Bekhogainsam decoction

Bekhogainsam decoction (BHID) is an ancient Chinese medicinal formula with a long history of application, created by Zhang Zhongjing, a prominent physician in the Eastern Han Dynasty. BHID is composed of Gypsumfibrosum, Anemarrhena asphodeloides Bunge, Panax ginseng C.A.Mey., *Glycyrrhiza* glabra L. and Oryzasativa L. seeds. Meng et al. (2020) utilized metabolomics combined with intestinal flora analysis to attempt to explain the mechanism of BHID in the treatment of DKD. They found that after oral administration of BHID (0.5 g/kg/d), there was a significant difference in the abundance of *Actinobacteria* phylum between the BHID-treated group and control groups. BHID may exert an inhibitory effect on chronic renal inflammation by regulating *Actinobacteria*. In addition, BHID was also found to be able to promote the growth of probiotics such as *Clostridiales* and *Peptococcus* and counteract bacterial dysbiosis in STZ-induced DKD mice. Moreover, it shows that BHID may be more effective in restoring the gut microbiota in DKD mice compared with metformin. This study also established an overall correlation between network pharmacology, metabolomics, and gut microbiota analysis, which falls in line with system biology.

#### 4.3.9 Jowiseungki decoction

Jowiseungki decoction (JSD) is a formula composed of Rheum palmatum L., Mirabilitum, and *Glycyrrhiza* glabra L. in a ratio of 4:2:1, which was also created by Zhang Zhongjing. There are relatively fewer studies on JSD for DKD. Meng et al. (2020) found that JSD (0.5 g/kg/d) was able to restore the intestinal flora composition of STZ-induced DKD mice, which in turn participated in the metabolic disorders and chronic inflammation of DKD, manifested by the reduction of fasting glucose, triglycerides, urinary albumin, and the reduction of renal tissue damage. The modified microbiota includes *Alphaproteobacteri*, *Atopobiaceaem*, *Acetatifactor*, *Butyricicoccus*, *Ker-stersia*, *Peptococcus*, and Coriobacteraceae*_UCG-002*.

### 4.4 Natural extracts

#### 4.4.1 Resveratrol

Resveratrol is a phytochemical of the stilbene family with anti-inflammatory, anti-oxidative stress, and anti-glycosylation properties, as well as a preventive effect on DM and its complications (Galiniak et al., 2018). According to Cai et al. (2020), resveratrol (10 mg/kg/d) reduced serum levels of urea nitrogen, creatinine, and 24-h UTP in db/db mice, along with decreased intestinal permeability and increased *Parabacteroides*, *Alistipes*, as well as *Bacteroides*, which are closely related to anti-inflammatory factors and exhibit anti-inflammatory properties. Similar results were also obtained by a fecal transplant from resveratrol-treated mice into db/db mice, suggesting that resveratrol may exert a nephroprotective effect by altering the composition of the intestinal flora. Resveratrol butyrate ester (RBE) is the esterification product of resveratrol and butyrate ester. A study randomly divided adenine-induced CKD rats into three groups to receive resveratrol (50 mg/L), low-dose RBE (25 mg/L), and high-dose RBE (50 mg/L), respectively. Both resveratrol and RBE were found to have nephroprotective effects. *Alistipes*, *Blautia*, and *Parabacteroides* which can produce SCFAs, were altered in the intestinal tract of CKD rats after the administration of resveratrol (Hsu et al., 2022). The high-dose RBE group showed an increase in the abundance of *Akkermansia*, *Blautia*, and *Enterococcus*, all of which are considered to be beneficial species (Cani and de Vos, 2017; Hanchi et al., 2018; Liu et al., 2021).

#### 4.4.2 Punicalagin

Punicalagin is the main component of pomegranates, which has powerful antioxidant and anti-inflammatory effects. In addition, studies have shown that punicalagin also has a protective effect against DKD (An et al., 2020). A study was conducted in which mice were randomly divided into control, DM, metformin (150 mg/kg/d), low-dose punicalagin (50 mg/kg/d), and high-dose punicalagin groups (100 mg/kg/d), and treated accordingly. The results showed that PU could amend the dysbiosis induced by high-fat diet feeding and increase the abundance of SCFAs as well as SCFAs-producing bacteria such as *Eubacterium_coprostanoligenes* and Lachnospiraceae in the intestine (Hua et al., 2022), thereby inhibiting the expression of inflammation-related genes and enhancing the intestinal barrier. The enhanced intestinal barrier could, in turn, counteract the elevated levels of LPS within the circulation due to bacterial ectopics. Additionally, punicalagin was also observed to be capable of countering insulin resistance induced by high-fat diets (Hua et al., 2022).

#### 4.4.3 Rehmannia glutinosa leaves total glycoside

As a primary component of Rehmannia glutinosa leaf, the pharmacological activity of Rehmannia glutinosa leaves total glycoside (DHY) has been widely studied. A study was conducted to investigate the mechanism of DHY against renal injuries; db/db mice were divided into model, metformin (250 mg/kg/d), irbesartan (50 mg/kg/d), Dihuangye total glycoside capsule (520 mg/kg/d), and DHY groups (2.6 g/kg/d). After the application of DHY, it was found that the blood urea nitrogen, creatinine, urinary trace protein, fasting glucose, and total cholesterol levels were significantly reduced. The alteration of intestinal flora showed an increase in Erysipelotrichaceae and *Acetatifactor* levels (Xu et al., 2020). It has been speculated that DHY may regulate glucolipid metabolism by altering intestinal flora and exhibiting kidney protection effects.

#### 4.4.4 Emodin

Emodin is the main active ingredient of Rheum palmatum L. Rheum palmatum L. enema has been widely used in the treatment of CKD, and studies about Emodin treatment have also been carried out. A study showed that emodin (1 mg/d) by colonic irrigation reduced urea levels, urinary protein amount, urea, and indole sulfate in CKD rats. Emodin was able to adjust the intestinal microbiota, reduce the abundance of *Clostridium* and increase *Lactobacillus* in 5/6 nephrectomy CKD rats (Zeng et al., 2016). The former was positively associated with uremic toxins production, while the latter was negatively correlated. Therefore, the renoprotective effect of Emodin may be mediated by the adjustment of the intestinal microbiota and the reduction of uremic toxins. However, emodin has a lower solubility and adheres to the intestinal mucosa for only a shorter period of time. Lu et al. (2021), on the other hand, modified Emodin by combining a nano-targeted drug delivery system with Emodin and creating the emodin-nanoparticle system (emodin-NP). They found that emodin-NP could regulate intestinal flora, enhance the intestinal barrier, reduce serum LPS, IL-6, and IL-1β levels, improve renal function and inhibit renal fibrosis in 5/6 nephrectomized rats. The abundance of *Saccharibacteria, Clostridiales, Butyricicoccus, and* Lachnospiraceae was increased after treatment with a low dose of emodin-NP (1.15 mg/kg every 2 days). In contrast, the abundance of *Clostridium*, *Aloprevotella*, *Romboutsia*, *Oscillibacter*, *Ruminococcus*, and *Turicibacter* was increased in the high dose emodin-NP (4.6 mg/kg, every 2 days) group, and treatment with emodin (4.6 mg/kg/d) increased *Streptococcus*. They concluded that emodin-NP administered once every 2 days was equivalent to emodin administered once daily. Nevertheless, emodin-NP is a 2-day enema, while the emodin group is a 1-day enema. Could the difference in the number of enemas also affect the final results obtained since each enema has a corresponding effect on the intestinal environment?

#### 4.4.5 Total flavones of abelmoschus manihot

The total flavones of Abelmoschus Manihot (TFA) are a main active component of a Chinese medicinal preparation, Huangkui capsules. Huangkui capsules have been widely used in clinical practice for the treatment of CKD, and their safety and effectiveness have been confirmed (Li et al., 2021). Tu et al. (2020) found that TFA (136 mg/kg/day) improved dysbiosis in chronic renal failure rats while inhibiting gut microbial-derived microinflammation to exert renoprotective effects. It was demonstrated by reducing *Bacteroidales* and *Lactobacillales* while increasing *Erysipelotrichales* and their corresponding metabolites. If different doses of TFA could be set in different groups, the study would be more enriched.

#### 4.4.6 Isoquercitrin

Isoquercitrin is a natural flavonoid that exists in various botanical drugs and can inhibit oxidative stress and inflammation (Shen et al., 2020). In addition, a study has demonstrated the role of isoquercitrin (80 mg/kg/d) in reducing the production of uremic toxins in the intestine of adenine-induced CKD mice. Interestingly, the mechanism of action may be through the reduction of indole production by inhibiting the activity of complex I in the bacterial membrane respiratory chain, which reduces tryptophan transport, rather than by modifying the composition of the gut microbiota (Wang et al., 2020). It would be more informative for the study results if isoquercitrin groupings of different doses and positive controls could be established.

#### 4.4.7 Curcumin

Curcumin is a natural polyphenolic compound and one of the major components of turmeric. Yang et al. (2015) found that Curcumin (500 mg/d) upregulated the levels of bacteria that favor the intestinal barrier in the gut of T2DM patients, such as *Bacteroides*, *Bifidobacterium,* and *Lactobacillus*. They hypothesized that the reduced plasma LPS and improved renal function observed in patients with T2DM might be due to Curcumin’s regulation of intestinal flora, which restored the epithelial barrier and reduced chronic inflammation triggered by LPS. Randomized controlled trials are needed to further elucidate the renal protective and gut microbiota-modulating effects of Curcumin.

#### 4.4.8 Fisetin

Fisetin is a natural compound widely present in various plants. Previous studies have shown the effect of Fisetin in slowing down the progression of renal fibrosis (Ren et al., 2021). The anti-fibrotic effect of Fisetin was further explored in a study. 16 S rRNA sequencing data showed that Fisetin (100 mg/kg) reduced the F/B ratio, reversed the adverse alteration of intestinal flora in hyperuricemia-induced CKD rats, and protect renal functions that might be mediated by the adjustment of gut microbiota composition and amino acid metabolism (Ren et al., 2021).

#### 4.4.9 Bupleurum polysaccharides

Bupleurum polysaccharides, a polysaccharide extracted from the Chinese herbal medicine Chai Hu, have been shown to be beneficial in animal models of diabetes (Pan et al., 2015). In a study, bupleurum polysaccharides were extracted from two different types of Bupleurums: Bupleurum chinense DC. and Bupleurum smithii var. Parvifolium, and denoted by BCP and BPs, respectively. Results have shown that the polysaccharides (60 mg/kg/d) extracted from both types increased the abundance of butyric acid-producing bacteria (*Roseburia* or *Eubacterium*) in the intestine of STZ-induced DKD mice, reduced blood creatinine and urinary protein levels, maintained the intestinal barrier and suppressed chronic inflammation possibly by enhancing the production of butyric acid (Pan et al., 2015).

#### 4.4.10 Cordyceps cicadae polysaccharides

Cordyceps cicadae polysaccharides (CCP) is a medicinal extract of the valuable Chinese medicine Cordyceps cicadae (a medicinal parasitic fungus) with anti-inflammatory and antioxidant properties (Sharma et al., 2015). CCP treatment can reduce blood creatinine and urine albumin levels and improve insulin resistance and glucose tolerance in STZ-induced DKD rats. It was also observed that CCP reduced the F/B ratio, increased the abundance of beneficial organisms closely related to intestinal barrier integrity, such as *Bacteroides*, *Lactobacillus*, *Bifidobacterium,* and *Akkermansia*, as well as beneficial organisms capable of producing SCFAs such as *Roseburia*, and decreased the abundance of pathogenic LPS-producing bacteria such as *Proteobacteria*. There was no significant difference in the composition of gut microbiota among CCP low-dose (75 mg/kg/d), medium-dose (150 mg/kg/d), and high-dose groups (300 mg/kg/d). Moreover, CCP is found to be able to protect intestinal barrier integrity and inhibit the progression of inflammation and renal fibrosis in DKD rats (Yang et al., 2020). It was found that the effects of CCP were comparable to those of dimethyl biguanide alone when the dose was 300 mg/kg.

According to the literature retrieved above, the most common changes in the intestinal flora associated with herbal medicine or natural extracts are changes in the abundance of *akkermansia*, *Lactobacillus*, and *bacteroidetes,* as well as changes in the F/B ratio. *Akkermansia* accounts for 1–4% of the total number of fecal microorganisms in healthy individuals from early life and plays an influential role in the physiopathology of the host. It was found that *akkermansia* abundance was significantly lower in CKD patients and was negatively correlated with IL-10 production (Li et al., 2019). *Akkermansia* has been shown to increase mucus thickness and intestinal mucosal barrier function (Ottman et al., 2017). And as discussed above, it can produce butyric acid, reduce intestinal mucosal damage, and decrease serum LPS levels as well (Belzer et al., 2017; Lopes et al., 2018). *Lactobacillus* was able to promote the production of SCFAs, improve the integrity of the intestinal barrier, and improve renal function by reducing renal injury and fibrosis-associated proteins (Niwa, 2013; Robles-Vera et al., 2018). In addition, it reduces oxidative stress and proinflammatory responses in the kidney and enhances immune responses (Huang et al., 2021). As organisms capable of producing SCFAs, *Bacteroides* possess certain anti-inflammatory and intestinal barrier protection properties (Wei et al., 2021).

Even though some of these studies have shown an effect of certain bacteria on some clinical parameters or metabolites of DKD, most were conducted in animal models, whereas clinical studies are scarce. Due to the fact that gut microbiota varies among species, the correlation between gut microbiota and DKD should be studied primarily in humans. Furthermore, most studies above observed changes in intestinal flora before and after obtaining the botanical drug and correlated laboratory indicators with these changes, which is less persuasive. A further consideration is whether the effect was caused by the intestinal flora and its metabolites or by the active substances of the herbal medicine transformed by the intestinal flora, which is more challenging to determine. To further elucidate the mechanisms through which herbal medicines regulate intestinal flora and their role in improving DKD, future studies may need to use relevant settings such as germ-free animals, antibiotic-treated animals, and setting up fecal transplant groups, and more rigorous experimental designs are needed.

## 5. Conclusion

**FIGURE 1:**
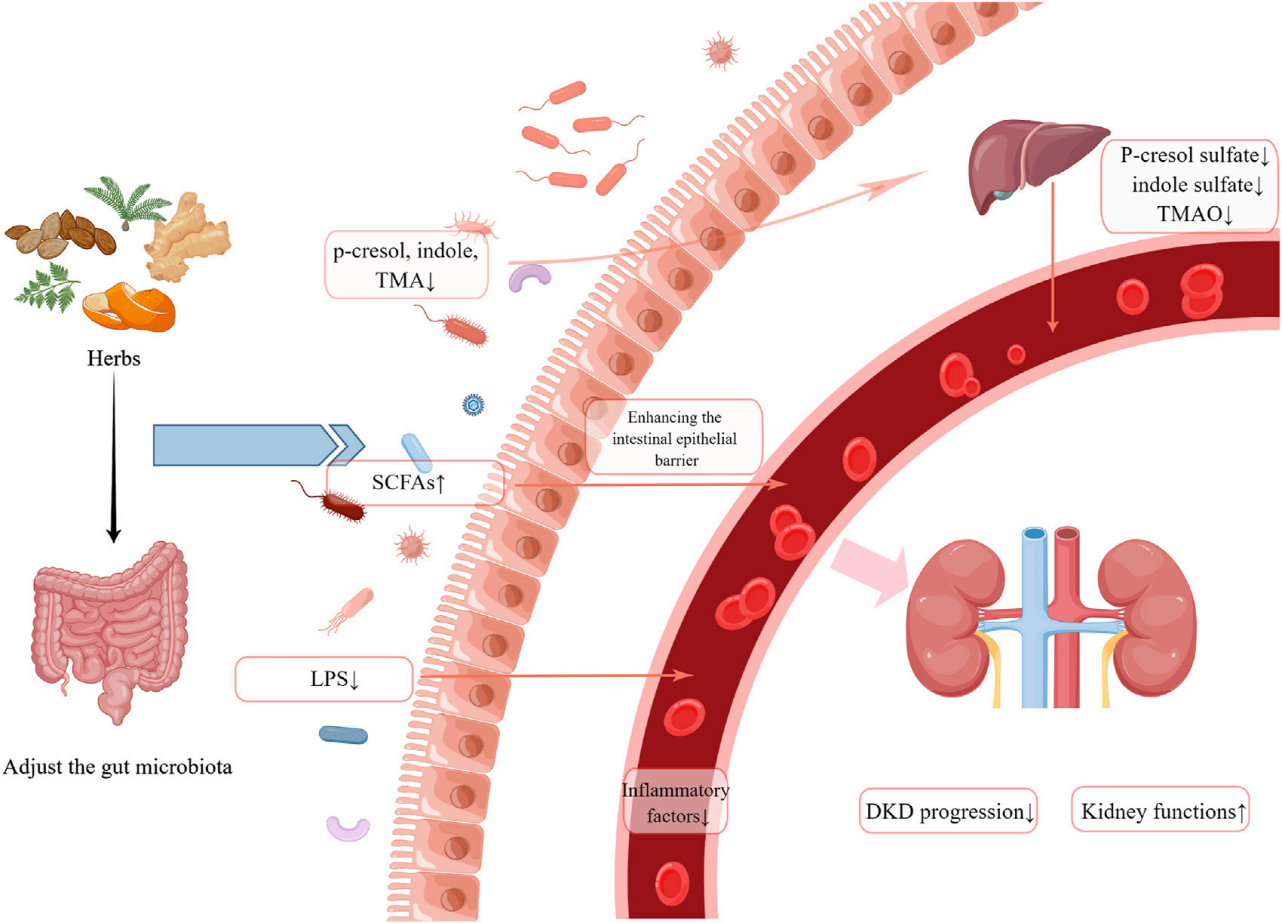
A summary of possible mechanisms by which herbal medicine may exert nephroprotective effects by influencing the gut microbiota. (Created by Figdraw).

A growing body of evidence suggests that the gut microbiota and their metabolites play a significant role in the pathogenesis of DM and DKD. In this review, we summarized the effects of botanical drugs, herbal pairs, formulas, and natural extracts on the intestinal flora of patients or animal models of kidney disease. The potential mechanisms of renoprotective effects by affecting the intestinal flora (Figure Figure1) have also been discussed. In accordance with the above studies, herbal or natural extracts have the following effects: modulating the composition of intestinal flora, particularly *Akkermansia*, *Lactobacillus*, and *Bacteroidetes*, as well as adjusting the F/B ratio; increasing the production of SCFAs and restoring the intestinal barrier; reducing the concentration of uremic toxins (p-cresol sulfate, indole sulfate, TMAO); inhibiting inflammation and oxidative stress. However, the number of such studies is still relatively low, and the limitation of this paper is the low inclusion of literature. Future studies may focus on the potential mechanisms by which herbal medicine protects renal function *via* its action on the gut-kidney axis. Understanding the role of botanical drugs in alleviating kidney injuries and regulating gut microbiota can provide new approaches and ideas for the treatment of DKD.

## Abbreviations

DKD, diabetic kidney disease; DM, diabetes mellitus; CKD, chronic kidney disease; ESRD, end-stage renal disease; SCFAs, short-chain fatty acids; TMAO, trimethylamine N-oxide; TMA, trimethylamine; LPS, lipopolysaccharides; T2DM, type 2 diabetes mellitus; 24-h UTP, 24-h urine protein quantity; HFD, high-fat diet; STZ, streptozotocin; SYKFT, Shenyan Kangfu tablet; TSF, Tangshen Formula; QDTS, Qidi Tangshen granules; SQYSF, Shenqi Yanshen formula; QRXZF, Qingre Xiaozheng formula; JPYS, Jianpi Yishen decoction; SHYS, Sanhuang Yishen capsule; BHID, Bekhogainsam decoction; JSD, Jowiseungki decoction; RBE, Resveratrol butyrate ester; DHY, Rehmannia glutinosa leaves total glycoside; TFA, total flavones of Abelmoschus Manihot;

## References

[B1] Allcock G. H. Allegra M. Flower R. J. Perretti M. (2001). Neutrophil accumulation induced by bacterial lipopolysaccharide: Effects of dexamethasone and annexin 1. Clin. Exp. Immunol. 123 (1), 62–67. 10.1046/j.1365-2249.2001.01370.x 11167999 PMC1905950

[B2] Amon P. Sanderson I. (2017). What is the microbiome? Arch. Dis. Child. Educ. Pract. Ed. 102 (5), 257–260. 10.1136/archdischild-2016-311643 28246123

[B3] An X. Zhang Y. Cao Y. Chen J. Qin H. Yang L. (2020). Punicalagin protects diabetic nephropathy by inhibiting pyroptosis based on TXNIP/NLRP3 pathway. Nutrients 12 (5), 1516. 10.3390/nu12051516 32456088 PMC7284711

[B4] Andrade-Oliveira V. Amano M. T. Correa-Costa M. Castoldi A. Felizardo R. J. F. de Almeida D. C. (2015). Gut bacteria products prevent AKI induced by ischemia-reperfusion. J. Am. Soc. Nephrol. 26 (8), 1877–1888. 10.1681/ASN.2014030288 25589612 PMC4520159

[B5] Antza C. Stabouli S. Kotsis V. (2018). Gut microbiota in kidney disease and hypertension. Pharmacol. Res. 130, 198–203. 10.1016/j.phrs.2018.02.028 29496593

[B6] Bammens B. Verbeke K. Vanrenterghem Y. Evenepoel P. (2003). Evidence for impaired assimilation of protein in chronic renal failure. Kidney Int. 64 (6), 2196–2203. 10.1046/j.1523-1755.2003.00314.x 14633143

[B7] Barutta F. Bellini S. Corbetta B. Durazzo M. Gruden G. (2020). The future of diabetic kidney disease management: What to expect from the experimental studies? J. Nephrol. 33 (6), 1151–1161. 10.1007/s40620-020-00724-1 32221858

[B8] Belzer C. Chia L. W. Aalvink S. Chamlagain B. Piironen V. Knol J. (2017). Microbial metabolic networks at the mucus layer lead to diet-independent butyrate and vitamin B12 production by intestinal symbionts. mBio 8 (5), e00770. 10.1128/mBio.00770-17 28928206 PMC5605934

[B9] Cai K. Ma Y. Cai F. Huang X. Xiao L. Zhong C. (2022). Changes of gut microbiota in diabetic nephropathy and its effect on the progression of kidney injury. Endocrine 76 (2), 294–303. 10.1007/s12020-022-03002-1 35246764

[B10] Cai T. Ye X. Li R. Chen H. Wang Y. Yong H. (2020). Resveratrol modulates the gut microbiota and inflammation to protect against diabetic nephropathy in mice. Front. Pharmacol. 11, 1249. 10.3389/fphar.2020.01249 32973502 PMC7466761

[B11] Cani P. D. de Vos W. M. (2017). Next-Generation beneficial microbes: The case of akkermansia muciniphila. Front. Microbiol. 8, 1765. 10.3389/fmicb.2017.01765 29018410 PMC5614963

[B12] Chen Q. Ren D. Wu J. Yu H. Chen X. Wang J. (2021). Shenyan Kangfu tablet alleviates diabetic kidney disease through attenuating inflammation and modulating the gut microbiota. J. Nat. Med.-Tokyo. 75 (1), 84–98. 10.1007/s11418-020-01452-3 32997272

[B13] Chen W. Zhang M. Guo Y. Wang Z. Liu Q. Yan R. (2021). The profile and function of gut microbiota in diabetic nephropathy. Diabetes Metab. Syndr. Obes. 14, 4283–4296. 10.2147/DMSO.S320169 34703261 PMC8541750

[B14] Cummings J. H. Pomare E. W. Branch W. J. Naylor C. P. Macfarlane G. T. (1987). Short chain fatty acids in human large intestine, portal, hepatic and venous blood. Gut 28 (10), 1221–1227. 10.1136/gut.28.10.1221 3678950 PMC1433442

[B15] Daly K. Shirazi-Beechey S. P. (2006). Microarray analysis of butyrate regulated genes in colonic epithelial cells. DNA Cell Biol. 25 (1), 49–62. 10.1089/dna.2006.25.49 16405400

[B16] Dinan T. G. Cryan J. F. (2017). Gut-brain axis in 2016: Brain-gut-microbiota axis - mood, metabolism and behaviour. Nat. Rev. Gastroenterol. Hepatol. 14 (2), 69–70. 10.1038/nrgastro.2016.200 28053341

[B17] Du X. Liu J. Xue Y. Kong X. Lv C. Li Z. (2021). Alteration of gut microbial profile in patients with diabetic nephropathy. Endocrine 73 (1), 71–84. 10.1007/s12020-021-02721-1 33905112

[B18] Evenepoel P. Poesen R. Meijers B. (2017). The gut–kidney axis. Pediatr. Nephrol. 32 (11), 2005–2014. 10.1007/s00467-016-3527-x 27848096

[B19] Fang Q. Zheng B. Liu N. Liu J. Liu W. Huang X. (2021). Trimethylamine N-oxide exacerbates renal inflammation and fibrosis in rats with diabetic kidney disease. Front. Physiol. 12, 682482. 10.3389/fphys.2021.682482 34220546 PMC8243655

[B20] Feng Y. Cao G. Chen D. Vaziri N. D. Chen L. Zhang J. (2019). Microbiome–metabolomics reveals gut microbiota associated with glycine-conjugated metabolites and polyamine metabolism in chronic kidney disease. Cell. Mol. Life Sci. 76 (24), 4961–4978. 10.1007/s00018-019-03155-9 31147751 PMC11105293

[B21] Feng Y. Weng H. Ling L. Zeng T. Zhang Y. Chen D. (2019). Modulating the gut microbiota and inflammation is involved in the effect of Bupleurum polysaccharides against diabetic nephropathy in mice. Int. J. Biol. Macromol. 132, 1001–1011. 10.1016/j.ijbiomac.2019.03.242 30946910

[B22] Fernandez-Prado R. Esteras R. Perez-Gomez M. V. Gracia-Iguacel C. Gonzalez-Parra E. Sanz A. B. (2017). Nutrients turned into toxins: Microbiota modulation of nutrient properties in chronic kidney disease. Nutrients 9 (5), E489. 10.3390/nu9050489 PMC5452219 28498348

[B23] Flint H. J. Scott K. P. Louis P. Duncan S. H. (2012). The role of the gut microbiota in nutrition and health. Nat. Rev. Gastroenterol. Hepatol. 9 (10), 577–589. 10.1038/nrgastro.2012.156 22945443

[B24] Friedman E. A. (2009). Can the bowel substitute for the kidney in advanced renal failure? Curr. Med. Res. Opin. 25 (8), 1913–1918. 10.1185/03007990903069173 19558343

[B25] Fukagawa M. Watanabe Y. (2011). Role of uremic toxins and oxidative stress in chronic kidney disease. Ther. Apher. Dial. 15 (2), 119. 10.1111/j.1744-9987.2010.00881.x 21426499

[B26] Galiniak S. Aebisher D. Bartusik-Aebisher D. (2018). Health benefits of resveratrol administration. Acta Biochim. Pol. 66 (1), 13–21. 10.18388/abp.2018_2749 30816367

[B27] Gao X. Liu H. An Z. He Q. (2018). QiDiTangShen granules reduced diabetic kidney injury by regulating the phosphorylation balance of the tyrosine and serine residues of insulin receptor substrate 1. Evid. Based. Complement. Altern. Med. 2018, 2503849. 10.1155/2018/2503849 PMC6046148 30050584

[B28] Gao X. Liu X. Xu J. Xue C. Xue Y. Wang Y. (2014). Dietary trimethylamine N-oxide exacerbates impaired glucose tolerance in mice fed a high fat diet. J. Biosci. Bioeng. 118 (4), 476–481. 10.1016/j.jbiosc.2014.03.001 24721123

[B29] Gao Y. Yang R. Guo L. Wang Y. Liu W. J. Ai S. (2021). Qing-Re-Xiao-Zheng formula modulates gut microbiota and inhibits inflammation in mice with diabetic kidney disease. Front. Med. 8, 719950. 10.3389/fmed.2021.719950 PMC8481597 34604258

[B30] Gheith O. Farouk N. Nampoory N. Halim M. A. Al-Otaibi T. (2016). Diabetic kidney disease: World wide difference of prevalence and risk factors. J. Nephropharmacol. 5 (1), 49–56. 28197499 PMC5297507

[B31] Gill S. R. Pop M. Deboy R. T. Eckburg P. B. Turnbaugh P. J. Samuel B. S. (2006). Metagenomic analysis of the human distal gut microbiome. Science 312 (5778), 1355–1359. 10.1126/science.1124234 16741115 PMC3027896

[B32] Gryp T. Huys G. R. B. Joossens M. Van Biesen W. Glorieux G. Vaneechoutte M. (2020). Isolation and quantification of uremic toxin precursor-generating gut bacteria in chronic kidney disease patients. Int. J. Mol. Sci. 21 (6), 1986. 10.3390/ijms21061986 32183306 PMC7139965

[B33] Gryp T. Vanholder R. Vaneechoutte M. Glorieux G. (2017). p-Cresyl Sulfate. Toxins 9 (2), E52. 10.3390/toxins9020052 PMC5331431 28146081

[B34] Guan Y. Chen K. Quan D. Kang L. Yang D. Wu H. (2021). The combination of Scutellaria baicalensis Georgi and Sophora japonica L. Ameliorate renal function by regulating gut microbiota in spontaneously hypertensive rats. Front. Pharmacol. 11, 575294. 10.3389/fphar.2020.575294 33643031 PMC7907655

[B35] Guo F. Dai Q. Zeng X. Liu Y. Tan Z. Zhang H. (2021). Renal function is associated with plasma trimethylamine-N-oxide, choline, l-carnitine and betaine: A pilot study. Int. Urol. Nephrol. 53 (3), 539–551. 10.1007/s11255-020-02632-6 32945995

[B36] Han C. Jiang Y. Li W. Liu Y. (2021). Astragalus membranaceus and Salvia miltiorrhiza ameliorates cyclosporin A-induced chronic nephrotoxicity through the “gut-kidney axis”. J. Ethnopharmacol. 269, 113768. 10.1016/j.jep.2020.113768 33383113

[B37] Hanchi H. Mottawea W. Sebei K. Hammami R. (2018). The genus Enterococcus: Between probiotic potential and safety concerns-an update. Front. Microbiol. 9, 1791. 10.3389/fmicb.2018.01791 30123208 PMC6085487

[B38] Hasegawa S. Jao T. Inagi R. (2017). Dietary metabolites and chronic kidney disease. Nutrients 9 (4), 358. 10.3390/nu9040358 28375181 PMC5409697

[B39] Hsiao Y. P. Chen H. L. Tsai J. N. Lin M. Y. Liao J. W. Wei M. S. (2021). Administration of lactobacillus reuteri combined with Clostridium butyricum attenuates cisplatin-induced renal damage by gut microbiota reconstitution, increasing butyric acid production, and suppressing renal inflammation. Nutrients 13 (8), 2792. 10.3390/nu13082792 34444952 PMC8402234

[B40] Hsu C. Hou C. Chang C. Tain Y. (2022). Resveratrol butyrate ester protects adenine-treated rats against hypertension and kidney disease by regulating the gut–kidney Axis. Antioxidants 11 (1), 83. 10.3390/antiox11010083 PMC8772985 35052587

[B41] Hua Q. Han Y. Zhao H. Zhang H. Yan B. Pei S. (2022). Punicalagin alleviates renal injuryvia the gut-kidney axis in high-fat diet-induced diabetic mice. Food Funct. 13 (2), 867–879. 10.1039/D1FO03343C 34989745

[B42] Huang H. Li K. Lee Y. Chen M. (2021). Preventive effects of lactobacillus mixture against chronic kidney disease progression through enhancement of beneficial bacteria and downregulation of gut-derived uremic toxins. J. Agric. Food Chem. 69 (26), 7353–7366. 10.1021/acs.jafc.1c01547 34170659

[B43] Huang K. Su Y. Sun M. Huang S. (2018). Chinese herbal medicine improves the long-term survival rate of patients with chronic kidney disease in taiwan: A nationwide retrospective population-based cohort study. Front. Pharmacol. 9, 1117. 10.3389/fphar.2018.01117 30327604 PMC6174207

[B44] Hugon P. Dufour J. Colson P. Fournier P. Sallah K. Raoult D. (2015). A comprehensive repertoire of prokaryotic species identified in human beings. Lancet. Infect. Dis. 15 (10), 1211–1219. 10.1016/S1473-3099(15)00293-5 26311042

[B45] Ji C. Deng Y. Yang A. Lu Z. Chen Y. Liu X. (2020). Rhubarb enema improved colon mucosal barrier injury in 5/6 nephrectomy rats may associate with gut microbiota modification. Front. Pharmacol. 11, 1092. 10.3389/fphar.2020.01092 32848732 PMC7403201

[B46] Ji C. Li Y. Mo Y. Lu Z. Lu F. Lin Q. (2021). Rhubarb enema decreases circulating trimethylamine N-oxide level and improves renal fibrosis accompanied with gut microbiota change in chronic kidney disease rats. Front. Pharmacol. 12, 780924. 10.3389/fphar.2021.780924 34966280 PMC8710758

[B47] Ji C. Lu F. Wu Y. Lu Z. Mo Y. Han L. (2022). Rhubarb enema increasing short-chain fatty acids that improves the intestinal barrier disruption in CKD may Be related to the regulation of gut dysbiosis. Biomed. Res. Int. 2022, 1896781. 10.1155/2022/1896781 35097110 PMC8794667

[B48] Karbach S. H. Schönfelder T. Brandão I. Wilms E. Hörmann N. Jäckel S. (2016). Gut microbiota promote angiotensin II–induced arterial hypertension and vascular dysfunction. J. Am. Heart Assoc. 5 (9), e003698. 10.1161/JAHA.116.003698 27577581 PMC5079031

[B49] Kim M. Yang J. Jo S. (2021). Intestinal microbiota and kidney diseases. Kidney Res. Clin. Pract. 40 (3), 335–343. 10.23876/j.krcp.21.053 34233442 PMC8476297

[B50] Koppe L. Fouque D. (2017). Microbiota and prebiotics modulation of uremic toxin generation. Panminerva Med. 59 (2), 173–187. 10.23736/S0031-0808.16.03282-1 28001024

[B51] Koppe L. Pillon N. J. Vella R. E. Croze M. L. Pelletier C. C. Chambert S. (2012). p-Cresyl sulfate promotes insulin resistance associated with CKD. J. Am. Soc. Nephrol. 24 (1), 88–99. 10.1681/ASN.2012050503 PMC3537215 23274953

[B52] Kou J. Wu J. Yang H. He Y. Fang J. Deng Y. (2014). Efficacy and safety of shenyankangfu tablets for primary glomerulonephritis: Study protocol for a randomized controlled trial. Trials 15 (1), 479. 10.1186/1745-6215-15-479 25480673 PMC4289030

[B53] Larsen N. Vogensen F. K. van den Berg F. W. J. Nielsen D. S. Andreasen A. S. Pedersen B. K. (2010). Gut microbiota in human adults with type 2 diabetes differs from non-diabetic adults. PLoS One 5 (2), e9085. 10.1371/journal.pone.0009085 20140211 PMC2816710

[B54] Li F. Wang M. Wang J. Li R. Zhang Y. (2019). Alterations to the gut microbiota and their correlation with inflammatory factors in chronic kidney disease. Front. Cell. Infect. Microbiol. 9, 206. 10.3389/fcimb.2019.00206 31245306 PMC6581668

[B55] Li N. Tang H. Wu L. Ge H. Wang Y. Yu H. (2021). Chemical constituents, clinical efficacy and molecular mechanisms of the ethanol extract ofAbelmoschus manihot flowers in treatment of kidney diseases. Phytother. Res. 35 (1), 198–206. 10.1002/ptr.6818 32716080 PMC7891592

[B56] Li P. Chen Y. Liu J. Hong J. Deng Y. Yang F. (2015). Efficacy and safety of tangshen formula on patients with type 2 diabetic kidney disease: A multicenter double-blinded randomized placebo-controlled trial. PLoS One 10 (5), e0126027. 10.1371/journal.pone.0126027 25938778 PMC4418676

[B57] Li Y. J. Chen X. Kwan T. K. Loh Y. W. Singer J. Liu Y. (2020). Dietary fiber protects against diabetic nephropathy through short-chain fatty acid–mediated activation of G protein–coupled receptors GPR43 and GPR109A. J. Am. Soc. Nephrol. 31 (6), 1267–1281. 10.1681/ASN.2019101029 32358041 PMC7269358

[B58] Li Y. Su X. Gao Y. Lv C. Gao Z. Liu Y. (2020). The potential role of the gut microbiota in modulating renal function in experimental diabetic nephropathy murine models established in same environment. Biochim. Biophys. Acta. Mol. Basis Dis. 1866 (6), 165764. 10.1016/j.bbadis.2020.165764 32169506

[B59] Liabeuf S. Barreto D. V. Barreto F. C. Meert N. Glorieux G. Schepers E. (2010). Free p-cresylsulphate is a predictor of mortality in patients at different stages of chronic kidney disease. Nephrol. Dial. Transpl. 25 (4), 1183–1191. 10.1093/ndt/gfp592 19914995

[B60] Lin C. Wu V. Wu P. Wu C. (2015). Meta-analysis of the associations of p-cresyl sulfate (PCS) and indoxyl sulfate (IS) with cardiovascular events and all-cause mortality in patients with chronic renal failure. PLoS One 10 (7), e0132589. 10.1371/journal.pone.0132589 26173073 PMC4501756

[B61] Liu C. Ma R. Wang L. Zhu R. Liu H. Guo Y. (2017). Rehmanniae radix in osteoporosis: A review of traditional Chinese medicinal uses, phytochemistry, pharmacokinetics and pharmacology. J. Ethnopharmacol. 198, 351–362. 10.1016/j.jep.2017.01.021 28111216

[B62] Liu X. Mao B. Gu J. Wu J. Cui S. Wang G. (2021). Blautia-a new functional genus with potential probiotic properties? Gut Microbes 13 (1), 1–21. 10.1080/19490976.2021.1875796 PMC7872077 33525961

[B63] Lopes R. Balbino K. P. Jorge M. P. Ribeiro A. Q. Martino H. Alfenas R. (2018). Modulation of intestinal microbiota, control of nitrogen products and inflammation by pre/probiotics in chronic kidney disease: A systematic review. Nutr. Hosp. 35 (3), 722–730. 10.20960/nh.1642 29974784

[B64] Lu C. C. Ma K. L. Ruan X. Z. Liu B. C. (2018). Intestinal dysbiosis activates renal renin-angiotensin system contributing to incipient diabetic nephropathy. Int. J. Med. Sci. 15 (8), 816–822. 10.7150/ijms.25543 30008592 PMC6036087

[B65] Lu Z. Ji C. Luo X. Lan Y. Han L. Chen Y. (2021). Nanoparticle-mediated delivery of emodin via colonic irrigation attenuates renal injury in 5/6 nephrectomized rats. Front. Pharmacol. 11, 606227. 10.3389/fphar.2020.606227 33551808 PMC7858270

[B66] Lu Z. Zhong Y. Liu W. Xiang L. Deng Y. (2019). The efficacy and mechanism of Chinese herbal medicine on diabetic kidney disease. J. Diabetes Res. 2019, 2697672–2697714. 10.1155/2019/2697672 31534972 PMC6732610

[B67] Meijers B. Jouret F. Evenepoel P. (2018). Linking gut microbiota to cardiovascular disease and hypertension: Lessons from chronic kidney disease. Pharmacol. Res. 133, 101–107. 10.1016/j.phrs.2018.04.023 29715498

[B68] Meijers B. K. De Loor H. Bammens B. Verbeke K. Vanrenterghem Y. Evenepoel P. (2009). p-Cresyl sulfate and indoxyl sulfate in hemodialysis patients. Clin. J. Am. Soc. Nephrol. 4 (12), 1932–1938. 10.2215/CJN.02940509 19833905 PMC2798868

[B69] Meng X. Ma J. Kang A. N. Kang S. Y. Jung H. W. Park Y. (2020). A novel approach based on metabolomics coupled with intestinal flora analysis and network pharmacology to explain the mechanisms of action of bekhogainsam decoction in the improvement of symptoms of streptozotocin-induced diabetic nephropathy in mice. Front. Pharmacol. 11, 633. 10.3389/fphar.2020.00633 32508632 PMC7253635

[B70] Meng X. Ma J. Kang S. Y. Jung H. W. Park Y. (2020). Jowiseungki decoction affects diabetic nephropathy in mice through renal injury inhibition as evidenced by network pharmacology and gut microbiota analyses. Chin. Med.-UK. 15 (1), 24. 10.1186/s13020-020-00306-0 PMC7066842 32190104

[B71] Monteiro R. C. Berthelot L. (2021). Role of gut–kidney axis in renal diseases and IgA nephropathy. Curr. Opin. Gastroenterol. 37 (6), 565–571. 10.1097/MOG.0000000000000789 34482323

[B72] Mosterd C. M. Kanbay M. van den Born B. J. H. van Raalte D. H. Rampanelli E. (2021). Intestinal microbiota and diabetic kidney diseases: The role of microbiota and derived metabolites inmodulation of renal inflammation and disease progression. Best. Pract. Res. Clin. Endocrinol. Metab. 35 (3), 101484. 10.1016/j.beem.2021.101484 33546983

[B73] Niwa T. (2013). Targeting protein-bound uremic toxins in chronic kidney disease. Expert Opin. Ther. Targets 17 (11), 1287–1301. 10.1517/14728222.2013.829456 23941498

[B74] Ottman N. Geerlings S. Y. Aalvink S. de Vos W. M. Belzer C. (2017). Action and function of Akkermansia muciniphila in microbiome ecology, health and disease. Best. Pract. Res. Clin. Gastroenterol. 31 (6), 637–642. 10.1016/j.bpg.2017.10.001 29566906

[B75] Pahl M. V. Vaziri N. D. (2015). The chronic kidney disease - colonic Axis. Semin. Dial. 28 (5), 459–463. 10.1111/sdi.12381 25855516

[B76] Pan L. Weng H. Li H. Liu Z. Xu Y. Zhou C. (2015). Therapeutic effects of bupleurum polysaccharides in streptozotocin induced diabetic mice. PLoS One 10 (7), e0133212. 10.1371/journal.pone.0133212 26176625 PMC4503743

[B77] Peng L. Li Z. Green R. S. Holzman I. R. Lin J. (2009). Butyrate enhances the intestinal barrier by facilitating tight junction assembly via activation of AMP-activated protein kinase in caco-2 cell monolayers. J. Nutr. 139 (9), 1619–1625. 10.3945/jn.109.104638 19625695 PMC2728689

[B78] Ren Q. Cheng L. Guo F. Tao S. Zhang C. Ma L. (2021). Fisetin improves hyperuricemia-induced chronic kidney disease via regulating gut microbiota-mediated tryptophan metabolism and aryl hydrocarbon receptor activation. J. Agric. Food Chem. 69 (37), 10932–10942. 10.1021/acs.jafc.1c03449 34505780

[B79] Ren Q. Tao S. Guo F. Wang B. Yang L. Ma L. (2021). Natural flavonol fisetin attenuated hyperuricemic nephropathy via inhibiting IL-6/JAK2/STAT3 and TGF-β/SMAD3 signaling. Phytomedicine. 87, 153552. 10.1016/j.phymed.2021.153552 33994251

[B80] Ríos J. (2011). Chemical constituents and pharmacological properties ofPoria cocos. Planta Med. 77 (07), 681–691. 10.1055/s-0030-1270823 21347995

[B81] Robles-Vera I. Toral M. de la Visitacion N. Sanchez M. Romero M. Olivares M. (2018). The probiotic lactobacillus fermentum prevents dysbiosis and vascular oxidative stress in rats with hypertension induced by chronic nitric oxide blockade. Mol. Nutr. Food Res. 62 (19), e1800298. 10.1002/mnfr.201800298 30028078

[B82] Rossi M. Johnson D. W. Morrison M. Pascoe E. M. Coombes J. S. Forbes J. M. (2016). Synbiotics easing renal failure by improving gut microbiology (synergy): A randomized trial. Clin. J. Am. Soc. Nephrol. 11 (2), 223–231. 10.2215/CJN.05240515 26772193 PMC4741035

[B83] Saglimbene V. Palmer S. C. Ruospo M. Natale P. Maione A. Nicolucci A. (2018). The long-term impact of renin-angiotensin system (RAS) inhibition on cardiorenal outcomes (lirico): A randomized, controlled trial. J. Am. Soc. Nephrol. 29 (12), 2890–2899. 10.1681/ASN.2018040443 30420421 PMC6287867

[B84] Schulman G. Berl T. Beck G. J. Remuzzi G. Ritz E. Arita K. (2015). Randomized placebo-controlled EPPIC trials of AST-120 in CKD. J. Am. Soc. Nephrol. 26 (7), 1732–1746. 10.1681/ASN.2014010042 25349205 PMC4483576

[B85] Sedighi M. Razavi S. Navab-Moghadam F. Khamseh M. E. Alaei-Shahmiri F. Mehrtash A. (2017). Comparison of gut microbiota in adult patients with type 2 diabetes and healthy individuals. Microb. Pathog. 111, 362–369. 10.1016/j.micpath.2017.08.038 28912092

[B86] Sharma S. K. Gautam N. Atri N. S. (2015). Optimized extraction, composition, antioxidant and antimicrobial activities of exo and intracellular polysaccharides from submerged culture of Cordyceps cicadae. BMC Complement. Altern. Med. 15, 446. 10.1186/s12906-015-0967-y 26694071 PMC4689043

[B87] Shen Y. Zhang Q. Huang Z. Zhu J. Qiu J. Ma W. (2020). Isoquercitrin delays denervated soleus muscle atrophy by inhibiting oxidative stress and inflammation. Front. Physiol. 11, 988. 10.3389/fphys.2020.00988 32903465 PMC7435639

[B88] Snelson M. Kellow N. J. Coughlan M. T. (2019). Modulation of the gut microbiota by resistant starch as a treatment of chronic kidney diseases: Evidence of efficacy and mechanistic insights. Adv. Nutr. 10 (2), 303–320. 10.1093/advances/nmy068 30668615 PMC6416045

[B89] Sonnenburg J. L. Bäckhed F. (2016). Diet–microbiota interactions as moderators of human metabolism. Nature 535 (7610), 56–64. 10.1038/nature18846 27383980 PMC5991619

[B90] Stavropoulou E. Kantartzi K. Tsigalou C. Konstantinidis T. Romanidou G. Voidarou C. (2020). Focus on the gut-kidney Axis in health and disease. Front. Med. 7, 620102. 10.3389/fmed.2020.620102 PMC7859267 33553216

[B91] Su X. Yu W. Liu A. Wang C. Li X. Gao J. (2022). San-huang-yi-shen capsule ameliorates diabetic nephropathy in rats through modulating the gut microbiota and overall metabolism. Front. Pharmacol. 12, 808867. 10.3389/fphar.2021.808867 35058786 PMC8764181

[B92] Sun C. Chang S. Wu M. (2012). Uremic toxins induce kidney fibrosis by activating intrarenal renin–angiotensin–aldosterone system Associated epithelial-to-mesenchymal transition. PLoS One 7 (3), e34026. 10.1371/journal.pone.0034026 22479508 PMC3316590

[B93] Sun G. Yin Z. Liu N. Bian X. Yu R. Su X. (2017). Gut microbial metabolite TMAO contributes to renal dysfunction in a mouse model of diet-induced obesity. Biochem. Biophys. Res. Commun. 493 (2), 964–970. 10.1016/j.bbrc.2017.09.108 28942145

[B94] Takiishi T. Fenero C. I. M. Câmara N. O. S. (2017). Intestinal barrier and gut microbiota: Shaping our immune responses throughout life. Tissue Barriers 5 (4), e1373208. 10.1080/21688370.2017.1373208 28956703 PMC5788425

[B95] Tang W. H. W. Wang Z. Kennedy D. J. Wu Y. Buffa J. A. Agatisa-Boyle B. (2015). Gut microbiota-dependent TrimethylamineN -oxide (TMAO) pathway contributes to both development of renal insufficiency and mortality risk in chronic kidney disease. Circ. Res. 116 (3), 448–455. 10.1161/CIRCRESAHA.116.305360 25599331 PMC4312512

[B96] Tao S. Li L. Li L. Liu Y. Ren Q. Shi M. (2019). Understanding the gut–kidney axis among biopsy-proven diabetic nephropathy, type 2 diabetes mellitus and healthy controls: An analysis of the gut microbiota composition. Acta Diabetol. 56 (5), 581–592. 10.1007/s00592-019-01316-7 30888537

[B97] Thursby E. Juge N. (2017). Introduction to the human gut microbiota. Biochem. J. 474 (11), 1823–1836. 10.1042/BCJ20160510 28512250 PMC5433529

[B98] Tu Y. Fang Q. Sun W. Liu B. Liu Y. Wu W. (2020). Total flavones of Abelmoschus manihot remodels gut microbiota and inhibits microinflammation in chronic renal failure progression by targeting autophagy-mediated macrophage polarization. Front. Pharmacol. 11, 566611. 10.3389/fphar.2020.566611 33101025 PMC7554637

[B99] van Baar M. J. B. van Ruiten C. C. Muskiet M. H. A. van Bloemendaal L. Ijzerman R. G. van Raalte D. H. (2018). SGLT2 inhibitors in combination therapy: From mechanisms to clinical considerations in type 2 diabetes management. Diabetes Care 41 (8), 1543–1556. 10.2337/dc18-0588 30030256

[B100] Vanholder R. Schepers E. Pletinck A. Nagler E. V. Glorieux G. (2014). The uremic toxicity of indoxyl sulfate and p-cresyl sulfate: A systematic review. J. Am. Soc. Nephrol. 25 (9), 1897–1907. 10.1681/ASN.2013101062 24812165 PMC4147984

[B101] Vanholder R. Schepers E. Pletinck A. Nagler E. V. Glorieux G. (2014). The uremic toxicity of indoxyl sulfate and p-cresyl sulfate: A systematic review. J. Am. Soc. Nephrol. 25 (9), 1897–1907. 10.1681/ASN.2013101062 24812165 PMC4147984

[B102] Wang X. Zhao L. Ajay A. K. Jiao B. Zhang X. Wang C. (2019). QiDiTangShen granules activate renal nutrient-sensing associated autophagy in db/db mice. Front. Physiol. 10, 1224. 10.3389/fphys.2019.01224 31632286 PMC6779835

[B103] Wang Y. Li J. Chen C. Lu J. Yu J. Xu X. (2020). Targeting the gut microbial metabolic pathway with small molecules decreases uremic toxin production. Gut Microbes 12 (1), 1–19. 10.1080/19490976.2020.1823800 PMC7577114 33016221

[B104] Wang Y. Yu F. Li A. He Z. Qu C. He C. (2022). The progress and prospect of natural components in rhubarb (Rheum ribes L.) in the treatment of renal fibrosis. Front. Pharmacol. 13, 919967. 10.3389/fphar.2022.919967 36105187 PMC9465315

[B105] Wei H. Wang L. An Z. Xie H. Liu W. Du Q. (2021). QiDiTangShen granules modulated the gut microbiome composition and improved bile acid profiles in a mouse model of diabetic nephropathy. Biomed. Pharmacother. 133, 111061. 10.1016/j.biopha.2020.111061 33378964

[B106] Williams R. Karuranga S. Malanda B. Saeedi P. Basit A. Besançon S. (2020). Global and regional estimates and projections of diabetes-related health expenditure: Results from the International Diabetes Federation Diabetes Atlas, 9th edition. Diabetes Res. Clin. Pract. 162, 108072. 10.1016/j.diabres.2020.108072 9th edition 32061820

[B107] Winther S. A. øllgaard J. C. Tofte N. Tarnow L. Wang Z. Ahluwalia T. S. (2019). Utility of plasma concentration of trimethylamine N-oxide in predicting cardiovascular and renal complications in individuals with type 1 diabetes. Diabetes Care 42 (8), 1512–1520. 10.2337/dc19-0048 31123156 PMC7082641

[B108] Wong J. Piceno Y. M. Desantis T. Z. Pahl M. Andersen G. L. Vaziri N. D. (2014). Expansion of urease- and uricase-containing, indole- and p-cresol-forming and contraction of short-chain fatty acid-producing intestinal microbiota in ESRD. Am. J. Nephrol. 39 (3), 230–237. 10.1159/000360010 24643131 PMC4049264

[B109] Wu I. W. Hsu K. H. Lee C. C. Sun C. Y. Hsu H. J. Tsai C. J. (2011). p-Cresyl sulphate and indoxyl sulphate predict progression of chronic kidney disease. Nephrol. Dial. Transpl. 26 (3), 938–947. 10.1093/ndt/gfq580 PMC3042976 20884620

[B110] Xia F. Wen L. P. Ge B. C. Li Y. X. Li F. P. Zhou B. J. (2021). Gut microbiota as a target for prevention and treatment of type 2 diabetes: Mechanisms and dietary natural products. World J. Diabetes 12 (8), 1146–1163. 10.4239/wjd.v12.i8.1146 34512884 PMC8394227

[B111] Xiu-Hai S. U. Shu-Quan L. U. Wang X. Y. Tian F. S. (2011). Clinical observation of sanhuang yishen capsule to early diabetic nephropathy. J. Liaoning Univ. Traditional Chin. Med. 13 (01), 18–19. 10.13194/j.jlunivtcm.2011.01.20.suxh.023

[B112] Xu K. Xia G. Lu J. Chen M. Zhen X. Wang S. (2017). Impaired renal function and dysbiosis of gut microbiota contribute to increased trimethylamine-N-oxide in chronic kidney disease patients. Sci. Rep.-UK. 7 (1), 1445. 10.1038/s41598-017-01387-y PMC5431124 28469156

[B113] Xu Z. Dai X. Zhang Q. Su S. Yan H. Zhu Y. (2020). Protective effects and mechanisms of Rehmannia glutinosa leaves total glycoside on early kidney injury in db/db mice. Biomed. Pharmacother. 125, 109926. 10.1016/j.biopha.2020.109926 32028239

[B114] Yang C. Y. Chen T. W. Lu W. L. Liang S. S. Huang H. D. Tseng C. P. (2021). Synbiotics alleviate the gut indole load and dysbiosis in chronic kidney disease. Cells 10 (1), 114. 10.3390/cells10010114 33435396 PMC7826693

[B115] Yang G. Wei J. Liu P. Zhang Q. Tian Y. Hou G. (2021). Role of the gut microbiota in type 2 diabetes and related diseases. Metabolism. 117, 154712. 10.1016/j.metabol.2021.154712 33497712

[B116] Yang H. Xu W. Zhou Z. Liu J. Li X. Chen L. (2015). Curcumin attenuates urinary excretion of albumin in type II diabetic patients with enhancing nuclear factor erythroid-derived 2-like 2 (Nrf2) system and repressing inflammatory signaling efficacies. Exp. Clin. Endocrinol. Diabetes 123 (06), 360–367. 10.1055/s-0035-1545345 25875220

[B117] Yang J. Dong H. Wang Y. Jiang Y. Zhang W. Lu Y. (2020). Cordyceps cicadae polysaccharides ameliorated renal interstitial fibrosis in diabetic nephropathy rats by repressing inflammation and modulating gut microbiota dysbiosis. Int. J. Biol. Macromol. 163, 442–456. 10.1016/j.ijbiomac.2020.06.153 32592781

[B118] Yang T. Richards E. M. Pepine C. J. Raizada M. K. (2018). The gut microbiota and the brain–gut–kidney axis in hypertension and chronic kidney disease. Nat. Rev. Nephrol. 14 (7), 442–456. 10.1038/s41581-018-0018-2 29760448 PMC6385605

[B119] Yao S. Zheng S. Zhang C. Zhao C. He X. Xu W. (2018). Mulberry leaf tea alleviates diabetic nephropathy by inhibiting PKC signaling and modulating intestinal flora. J. Funct. Foods. 46, 118–127. 10.1016/j.jff.2018.04.040

[B120] Ye X. He J. Xu J. He X. Xia C. Yan Y. (2020). Undescribed morroniside-like secoiridoid diglycosides with α-glucosidase inhibitory activity from Corni Fructus. Phytochemistry 171, 112232. 10.1016/j.phytochem.2019.112232 31911266

[B121] Zeng Y. Dai Z. Lu F. Lu Z. Liu X. Chen C. (2016). Emodinvia colonic irrigation modulates gut microbiota and reduces uremic toxins in rats with chronic kidney disease. Oncotarget 7 (14), 17468–17478. 10.18632/oncotarget.8160 27003359 PMC4951226

[B122] Zhang L. Yang L. Shergis J. Zhang L. Zhang A. L. Guo X. (2019). Chinese herbal medicine for diabetic kidney disease: A systematic review and meta-analysis of randomised placebo-controlled trials. BMJ Open 9 (4), e025653. 10.1136/bmjopen-2018-025653 PMC6501976 31048437

[B123] Zhang L. Zhang T. Li Y. Xiong W. (2022). Shenqi Yanshen Formula (SQYSF) protects against chronic kidney disease by modulating gut microbiota. Bioengineered 13 (3), 5625–5637. 10.1080/21655979.2021.2023789 35184655 PMC8974014

[B124] Zhang Z. Yang L. Wan Y. Liu C. Jiang S. Shang E. (2021). Integrated gut microbiota and fecal metabolomics reveal the renoprotective effect of Rehmanniae Radix Preparata and Corni Fructus on adenine-induced CKD rats. J. Chromatogr. B Anal. Technol. Biomed. Life Sci. 1174, 122728. 10.1016/j.jchromb.2021.122728 33975272

[B125] Zhao T. Zhang H. Yin X. Zhao H. Ma L. Yan M. (2020). Tangshen formula modulates gut microbiota and reduces gut-derived toxins in diabetic nephropathy rats. Biomed. Pharmacother. 129, 110325. 10.1016/j.biopha.2020.110325 32535383

[B126] Zheng L. Chen S. Wang F. Huang S. Liu X. Yang X. (2020). Distinct responses of gut microbiota to jian-pi-yi-shen decoction are associated with improved clinical outcomes in 5/6 nephrectomized rats. Front. Pharmacol. 11, 604. 10.3389/fphar.2020.00604 32435197 PMC7219274

[B127] Zhong C. Dai Z. Chai L. Wu L. Li J. Guo W. (2021). The change of gut microbiota-derived short-chain fatty acids in diabetic kidney disease. J. Clin. Lab. Anal. 35 (12), e24062. 10.1002/jcla.24062 34689373 PMC8649351

[B128] Zhu B. Wang X. Li L. (2010). Human gut microbiome: The second genome of human body. Protein Cell 1 (8), 718–725. 10.1007/s13238-010-0093-z 21203913 PMC4875195

